# Multi-modal Neuroimaging Phenotyping of Mnemonic Anosognosia in the Aging Brain

**DOI:** 10.1038/s43856-024-00497-9

**Published:** 2024-04-05

**Authors:** Elisenda Bueichekú, Ibai Diez, Geoffroy Gagliardi, Chan-Mi Kim, Kayden Mimmack, Jorge Sepulcre, Patrizia Vannini

**Affiliations:** 1 Gordon Center for Medical Imaging, Department of Radiology, Massachusetts General Hospital, Harvard Medical School, Boston, MA USA; 2 Athinoula A. Martinos Center for Biomedical Imaging, Department of Radiology, Massachusetts General Hospital, Harvard Medical School, Charlestown, MA USA; 3 Department of Neurology, Brigham and Women’s Hospital, Harvard Medical School, Boston, MA USA; 4 Department of Radiology, Yale PET Center, Yale Medical School, Yale University, New Haven, CT USA; 5 Department of Neurology, Massachusetts General Hospital, Harvard Medical School, Boston, MA USA

**Keywords:** Consciousness, Functional magnetic resonance imaging, Positron-emission tomography, Predictive markers, Alzheimer's disease

## Abstract

**Background:**

Unawareness is a behavioral condition characterized by a lack of self-awareness of objective memory decline. In the context of Alzheimer’s Disease (AD), unawareness may develop in predementia stages and contributes to disease severity and progression. Here, we use in-vivo multi-modal neuroimaging to profile the brain phenotype of individuals presenting altered self-awareness of memory during aging.

**Methods:**

Amyloid- and tau-PET (*N* = 335) and resting-state functional MRI (*N* = 713) imaging data of individuals from the Anti-Amyloid Treatment in Asymptomatic Alzheimer’s Disease (A4)/Longitudinal Evaluation of Amyloid Risk and Neurodegeneration (LEARN) Study were used in this research. We applied whole-brain voxel-wise and region-of-interest analyses to characterize the cortical intersections of tau, amyloid, and functional connectivity networks underlying unawareness in the aging brain compared to aware, complainer and control groups.

**Results:**

Individuals with unawareness present elevated amyloid and tau burden in midline core regions of the default mode network compared to aware, complainer or control individuals. Unawareness is characterized by an altered network connectivity pattern featuring hyperconnectivity in the medial anterior prefrontal cortex and posterior occipito-parietal regions co-locating with amyloid and tau deposition.

**Conclusions:**

Unawareness is an early behavioral biomarker of AD pathology. Failure of the self-referential system in unawareness of memory decline can be linked to amyloid and tau burden, along with functional network connectivity disruptions, in several medial frontal and parieto-occipital areas of the human brain.

## Plain language summary

Lack of self-awareness of cognitive changes, such as memory decline, occurs in people who later go on to develop Alzheimer’s disease. In the present study, we investigated various characteristics of the brains of people who were unaware they were experiencing memory loss and likely to develop Alzheimer’s disease due to their age. We identified individuals with low performance in memory tests and a lack of sense of their memory decline. Compared to aware individuals, they had more deposits of proteins known to be present at higher levels in people with Alzheimer’s disease. The results of this investigation suggest that unawareness of memory decline is an early behavioral sign that a person might develop Alzheimer’s disease. This knowledge might enable such people to be more easily identified in the future, and treatments to be started sooner.

## Abstract (web summary)

Bueichekú et al. use multimodal in vivo neuroimaging to investigate the brain characteristics of individuals presenting unawareness of memory loss who are at risk of Alzheimer’s disease due to age. They find unawareness of memory decline is an early behavioral sign that a person might develop Alzheimer’s disease.

## Introduction

Unawareness of memory loss^1,2^, is the inability to recognize memory impairments and the incapacity of updating and integrating novel information, producing a mismatch between the acquired knowledge and stored autobiographical experiences and the ongoing perceptual inputs, namely a petrified self ^3–6^. In this work, we investigate the brain phenotype of individuals presenting lower self-awareness of subtle memory decline and refer to this phenomenon as unawareness. In the context of Alzheimer’s disease (AD), unawareness of memory loss is a common symptom with a prevalence estimated to range between 20-80%^7,8^. It appears in pre-clinical to prodromal stages and is considered independent from primary cognitive deficits (i.e., attention, memory, executive functioning)^1,6,9–11^. It has been related to the deterioration of metacognition or self-monitoring^10^ and contributes to disease severity, symptomatology worsening, such as disinhibition and dangerous behavior (as unaware individuals are not aware of their limitations), aggravating mood disorders like depression and anxiety, and, in late stages, to loss of self-identity. Unawareness of cognitive deficits increases caregiver burden^3,9–17^ and earlier institutionalization. Previous research has demonstrated that the phenomenon is associated with a higher level of AD neuropathology, and it is also a good predictor of clinical progression^11,12,15,18–24^. In all, unawareness is clinically relevant as an indicator of a patient’s reliability to report self-dysfunction^21^. At the research level, it could be considered a behavioral marker of AD pathology presence in cognitively normal individuals and individuals showing subtle cognitive decline.

Positron emission tomography (PET) imaging research has linked greater AD-related changes to lower awareness of memory performance in specific AD-affected brain structures. That is, in studies using fluorodeoxyglucose-PET (FDG-PET), decreased metabolism in the posterior cingulate cortex has been consistently related to unawareness^14,25–28^. Lower awareness has been linked to increased global amyloid^11,20^ and more tau in medial temporal lobe (MTL)^29,30^. Interestingly, postmortem studies indicate unawareness in elderly adults, with or without dementia, is solely linked to one of the pathological events associated with AD: the presence of tau neurofibrillary tangles (NFTs)^24^. Structural and functional neuroimaging has linked unawareness to several cortical systems in the human brain^31,32^. Previous studies have described brain atrophy in MTL structures, lateral and medial prefrontal cortex, anterior cingulate cortex, insula, and fusiform gyrus in unaware subjects^33–36^. At the functional level, it features dysfunction of the frontal lobe or midline temporal cortex structures^30,37,38^ and disconnection within the subsystems of the default mode network (DMN) and between this network and MTL structures^27,28,39^. Consistent with AD pathology, unawareness in early stages is associated with a functional decline of midline structures and, in later stages, with parietotemporal and frontotemporal systems failures^38^. AD-related amyloid and tau pathology initially affect topographically distinct circuits, with tau first damaging brain stem nuclei and medial temporal cortex. In contrast, amyloid spreads initially in midline areas of the neocortex. Due to this distinct initial pattern of accumulation, some authors suggest that early pathology is independent, while in later disease stages, it is thought that amyloid drives tau pathology in arresting neocortical regions. In parallel, it has been observed that in preclinical stages, tau burden closely tracks memory changes and predicts future cognitive decline. In unawareness, it remains unknown whether single factors -such as tau aggregation- or multiple AD-related pathological players – like the co-occurrence of amyloid deposition and tau aggregation – coordinate together in vivo to produce the emergence of unawareness in the preclinical stages of AD.

The aim of the present research is to describe from a multi-modal neuroimaging analytical approach the brain phenotype underlying unawareness in the aging brain. We hypothesize that adults who present unawareness show a distinct pattern of amyloid and tau deposition, prospective pathology spreading, and specific functional disconnectomic profiles when compared to adults who are aware of subtle memory decline and to control participants. In this research, we observe that unawareness of memory decline in older individuals is marked by elevated amyloid and tau burden in midline core regions of the default mode network, coupled with functional network hyperconnectivity.

## Methods

### Participants

Imaging data of 1725 participants from the Anti-Amyloid Treatment in Asymptomatic Alzheimer’s (A4) and Longitudinal Evaluation of Amyloid Risk and Neurodegeneration (LEARN) studies was retrieved^40^. The A4 study is a three-year, placebo-controlled, randomized clinical trial conducted in 1000 older individuals with evidence of amyloid accumulation on screening PET scans, that test will whether anti-amyloid treatment can slow the rate of cognitive decline on a composite measure of sensitive neuropsychological tests^40^ [ClinicalTrials.gov number: NCT02008357]. The LEARN cohort comprises participants from the observational arm of the A4 study^40^. The main outcomes of the A4 study have been published in^41^. We only use baseline imaging and behavioral data from the A4/LEARN studies in the present study. Participants were distributed into two groups: i) an amyloid- and tau-PET sample: consisting of 447 participants, with final sample *N* = 335 (Table 1); ii) an fMRI sample: with a total of 1278 participants, with a final sample *N* = 713 (Table 2) (see Supplementary Fig. 1). Participants from the PET sample and the fMRI sample are independent (i.e., samples are not mixed throughout the analysis). Functional MRI data from the PET sample has not been used in the current study to avoid circularity. A4/LEARN participants underwent a series of screening visits to determine their eligibility^42^ (screening visit 1: collection of demographic information, apolipoprotein E genotyping, cognitive testing, and clinical assessments to determine eligibility to proceed to screening visit 2; screening visit 2: amyloid-PET imaging). One of the eligibility criterions is the amyloid status, which is determined using Florbetapir amyloid imaging. According to amyloid status, A4/LEARN participants are sub-classified into two groups: amyloid elevated participants (in our PET sample: 214 individuals; in our fMRI sample: 352 individuals), or not elevated amyloid participants (in our PET sample: 43 individuals; in our fMRI sample: 234 individuals). In our PET and fMRI sample 78 and 127 individuals are not assigned to either group respectively. In the present work the amyloid status classification was not used throughout the analysis because we used a continuous variable strategy. Screening included demographic, family history, lifestyle questionnaires, cognitive testing, functional questionnaires, and medical screening. Participants with a Clinical Dementia Rating (CDR) global score of zero, Mini-Mental State Examination (MMSE) score of 25 to 30, and Logical Memory delayed recall (LM)^43^ score of 6 to18 were eligible to proceed to imaging procedures. As reported in^42^ participants with high LM scores (>1.5 standard deviations (SD) above norms in this age range) were excluded from the study. Institutional Review Board (IRB) approval was secured at the participating sites. All A4 and LEARN participants provided informed consent prior to any research step in compliance with local IRB and provided permission to share their de-identified data to advance the quest to find a successful treatment for Alzheimer’s disease. Because the current study reuses data from the A4 study, we did not seek specific IRB approval for our study.

**Table 1:** Demographics of the PET sample

	Aware	Unaware	Complainer	Control	Group difference
N (% Females)	72 (45.8%)	25 (40 %)	151 (64.9 %)	87 (62.1 %)	
Mean age (SD)	73.10 (4.85)	72.98 (3.85)	70.36 (4.43)	70.75 (4.30)	H(3) = 23.69, *p* = 2.91e−5 *** ε^2^ = 0.07
Mean education (SD)	17 (2.64)	15.68 (1.84)	16.07 (2.68)	16.18 (3.22)	H(3) = 9.84, *p* = 0.02 * ε^2^ = 0.03
Mean MMSE (SD)	28.25 (1.63)	28.4 (1.54)	28.78 (1.15)	28.85 (1.21)	H(3) = 7.67, *p* = 0.053 ε^2^ = 0.02
Mean MACQ (SD)	27.46 (2.43)	21.80 (2.42)	27.19 (2.18)	21.53 (2.56)	H(3) = 32.03, *p* = 5.17e−7 *** ε^2^ = 0.68
Mean Imm. logic memory (SD)	11.57 (3.10)	12.64 (3.41)	13.62 (3.13)	13.33 (3.17)	
Mean Del. logic memory (SD)	9.78 (3.31)	10.64 (3.39)	12.38 (3.30)	12.26 (3.16)	H(3) = 32.03, *p* = 5.17e−7 *** ε^2^ = 0.09
Mean FCSRT free (SD)	22.46 (4.33)	21.20 (3.33)	31.03 (4.21)	31.45 (4.10)	F(3) = 109.8, *p* = 2e−16 *** η^2^ = 0.50
Mean FCSRT cued (SD)	24.53(4.01)	25.76(3.27)	16.50(4.12)	16.10(4.16)	F(3) = 101.7, *p* = 2e−16 *** η^2^ = 0.48
Mean FCSRT total (SD)	46.99 (1.95)	46.96 (0.84)	47.53 (0.80)	47.55 (0.66)	

*N* = 335 individuals. Legend: *p < 0.05, **p < 0.005, ***p < 0.001.

**Table 2:** Demographics of the fMRI sample

	Aware	Unaware	Complainer	Control	Group difference
N (% Females)	129 (50.4%)	74 (33.8%)	298 (67.8%)	212 (69.7%)	
Mean age (SD)	71.99 (4.91)	72.45 (5.06)	70.89 (4.42)	70.34 (4.31)	H(3) = 16.39, *p* = 9.42e−4*** ε^2^ = 0.02
Mean education (SD)	17.4 (2.93)	16.95 (2.7)	16.38 (2.59)	16.98 (2.73)	H(3) = 18.05, *p* = 4.29e−4*** ε^2^ = 0.01
Mean MMSE (SD)	28.67 (1.34)	28.18 (1.45)	28.90 (1.15)	28.92 (1.19)	H(3) = 19.37, *p* = 1.75e−4*** ε^2^ = 0.03
Mean MACQ (SD)	27.97 (2.65)	21.34 (2.80)	27.43 (2.38)	21.18 (3.01)	H(3) = 518.66, *p* = 2.2e−16*** ε^2^ = 0.73
Mean Imm. logic memory (SD)	11.19 (3.15)	11.50 (2.85)	13.52 (3.40)	13.72 (3.27)	
Mean Del. logic memory (SD)	9.57 (3.10)	9.99 (3.09)	12.27 (3.30)	12.69 (3.11)	H(3) = 99.172, *p* = 2.203−16*** ε^2^ = 0.14
Mean FCSRT free (SD)	23.12 (4.98)	23.05 (4.89)	31.10 (4.08)	31.00 (3.77)	F(3) = 169.4, *p* = 2e−16*** η^2^ = 0.42
Mean FCSRT cued (SD)	23.82(4.75)	23.95(4.79)	16.32 (3.96)	16.43 (3.65)	F(3) = 160.5, *p* = 2e−16***
Mean FCSRT total (SD)	46.94 (1.08)	47.00 (1.30)	47.42 (0.75)	47.43 (0.79)	η^2^ = 0.40

*N* = 713 individuals. Legend: *p < 0.05, **p < 0.005, ***p < 0.001.

### Experimental group definition

**Fig. 1:**
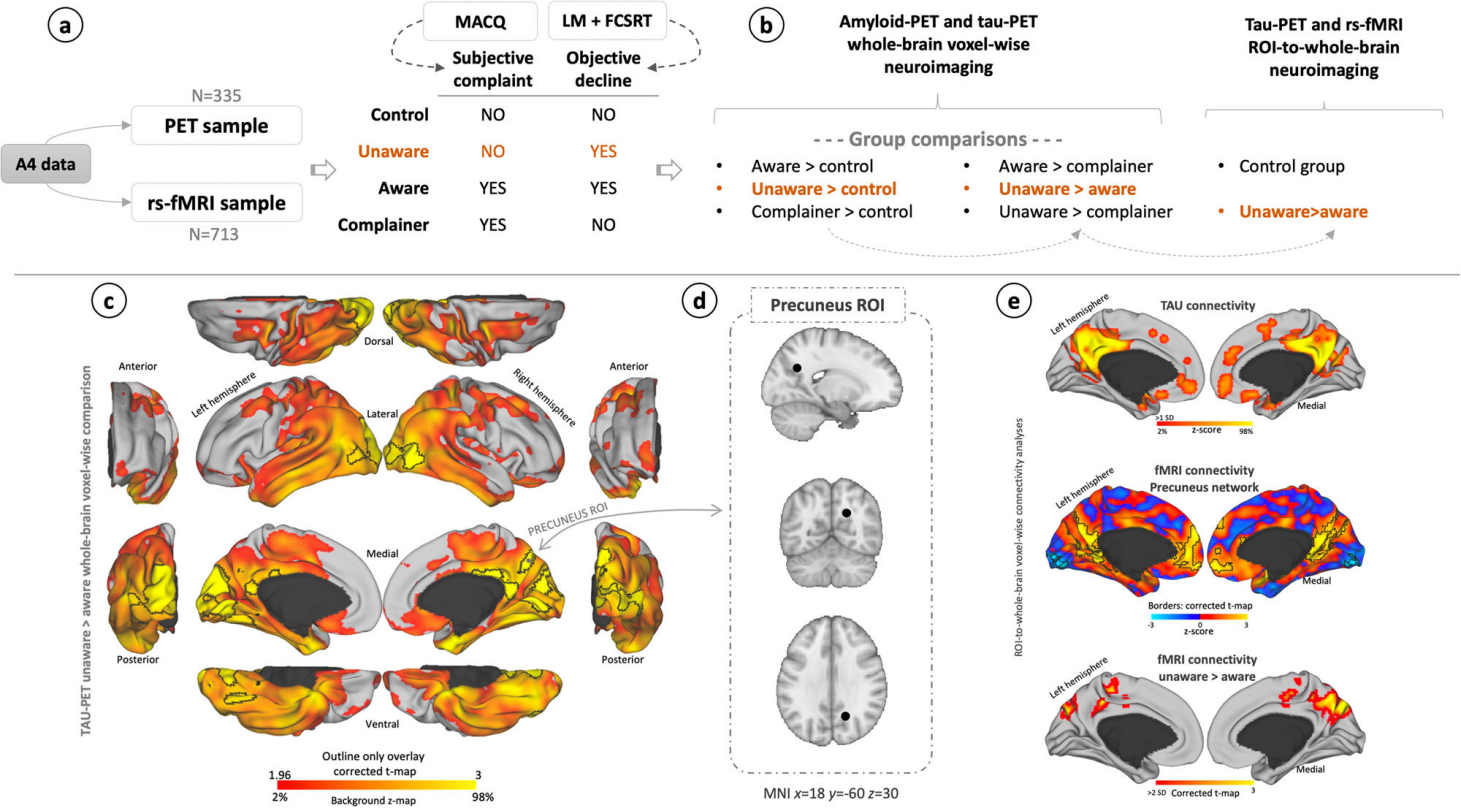
Methods overview. **a** Summarizing schema of the data used in the current study and experimental group definition. **b** Main neuroimaging analyses. **c** Brain projection example of group-level whole-brain voxel-wise comparisons. In this case, the tau-PET images of the individuals with unawareness were compared to the aware group. The outline border shows the results corrected for multiple comparisons. **d** Brain location example of the region of interest (ROI) derived from the comparison unaware > aware (the complete list of ROIs can be found in the Supplementary Table 1). In this case, the precuneus ROI and the subsequent analysis from this region are showed. The coordinates are in the Montreal Neurological Institute (MNI) space. **e** Brain projection examples of the whole-brain voxel-wise connectivity analysis done in the control group data for investigating tau spreading (upper brain map), functional connectivity with resting-state fMRI (middle brain map), as well as the group comparisons in functional connectivity (lower brain map).

Four subgroups were defined to investigate the brain phenotypic characteristic of adults with lower self-awareness (aware, unaware, subjective complainer, and control groups; Fig. 1a). Classification of participants into groups was based on objective and subjective memory assessments. We used neuropsychological tests to assess participants’ performance on memory system and to evaluate objective memory decline: the free and cued selective reminding (FCSRT) test^44^ and the LM test. A participant was classified as having memory decline when performance in both tests was below population norms. The delayed score from the LM assesses episodic memory and is used in combination with years of education to determine memory impairments (a score equal to or less than 8 for 16 years of education, a score of 4 for 8 to 15 years of education, and a score of 2 for 0 to 7 years of education). FCSRT offers a controlled learning setting to a reliable metric that englobes episodic memory encoding, recording, and retrieving processes. A score equal to or inferior to 24 in free recall and 44 in total recall in FCSRT indicates objective memory problems. For assessing subjective memory performance, the Memory Assessment Clinic Questionnaire (MACQ)^45^ was used. The MACQ is a brief self-reported questionnaire composed by 5 questions, like: recalling where you have put objects (such as keys) in your home or office, or remembering specific facts from a newspaper or magazine article you have just finished reading. Participants must reply to these questions in relation to when they were in high school or college (specific instruction: as compared to when you were in high school or college, how would you describe your ability to perform the following tasks involving your memory). The questionnaire includes a final and more general question: in general, how would you describe your memory as compared to when you were in high school. For all 6 questions, it uses a Likert-rating scale (i.e., much better now, somewhat better now, about the same, somewhat poorer now, much poorer now). The MACQ generates a score (range 7-35) that quantifies degree of memory complaint. Overall, this questionnaire targets age-related changes in that the subject is asked to rate current abilities compared to past abilities^45^. A participant is considered to have subjective memory complaints when the score is equal to or superior to 25^45^. Subsequently, the aware group is characterized by having both memory complaints and objective memory impairments. The individuals in the unaware group have objective memory loss, but these participants do not demonstrate subjective memory complains. Contrary to the unaware group, the complainer group shows subjective memory complaints but does not have objective memory impairments. Finally, the control group does not have subjective or objective memory problems. In this study, we focus on investigating the neurobehavioral characteristics of the unaware group, compared to the aware, the complainer, and the control group, which cover the spectrum of possible aging profiles.

### Between groups comparisons with behavioral data

We used the Cramer-von Mises normality test to assess the sample distribution of age, years of education, MMSE, and the subjective (MACQ) and objective memory scores (LM, free and cued FCSRT). For age, years of education, and MMSE, a non-parametric approach with a Kruskal-Wallis’ test was used to evaluate group differences. For assessing between-groups differences in MACQ and LM delayed score, as the data distribution did not follow a normal curve, we conducted a Kruskal-Wallis’ test followed by a post-hoc Dunn’s test, with a Benjamini-Hochberg multiple comparison correction in both statistical tests. We conducted a one-way ANOVA and a post-hoc Tukey’s test for free and cued FCSRT analyses. Dunnett’s test for planned group comparisons was also used for FCSRT tests. We used the R package for all statistical analyses (https://www.R-project.org/)^46^.

### PET, MRI, and fMRI acquisition and preprocessing

A4 is a multicenter imaging study acquiring data across North America. Detailed acquisition information should be found in A4 website within the IDA repository (https://ida.loni.usc.edu/). The following acquisition parameters were described in the study protocols or recovered from the NiFTI images.

#### PET acquisition

Amyloid PET imaging was collected 50 to 70 minutes post-injection (18-florbetapir [18F] FBP-PET). Amyloid images were reconstructed in 4 × 5 minute frames, with the exception of some sites that reconstructed the data in 50 to 70 minutes data, in a single frame. Tau PET imaging was collected 80 to 110 minutes post-injection ([18 F] FTP-PET). Tau images were reconstructed in 6 × 5 minute frames with the exception of some sites that reconstructed the data in 80 to 110 minutes data, in a single frame.

#### Structural and functional MRI acquisitions

 MRI scanners used in the A4 study are General Electrics, Siemens, Phillips Medical Systems or Philips Healthcare. High resolution 1 mm isotropic 3D T1-weighted structural images and resting state functional MRI were used in this study. Resting state functional MRI was used to measure changes in blood oxygenation level dependent (BOLD) T2* signal while the participants remained still with their eyes open. Gradient echo or gradient echo planar imaging (EPI) sequences with the following parameters were acquired: 3000 ms TR; 30 ms TE; 80- or 90-degrees flip angle; 3 mm isotropic voxels.

#### Positron emission tomography preprocessing

 FMRIB Software Library v6.0.4 (FSL; https://fsl.fmrib.ox.ac.uk/fsl/fslwiki/) and FreeSurfer v6 (https://surfer.nmr.mgh.harvard.edu/) were used for PET preprocessing performing the following steps: FreeSurfer preprocessing of the structural image, co-registration and average of PET frames, rigid body transformation between PET and structural MRI image, computation of standardized uptake value ratio (SUVr), partial volume correction (using FreeSurfer three-compartment model - Müller-Gärtner (MG) method), transformation to standard MNI152 space and spatial smoothing with an isotropic Gaussian kernel of 8 mm full width at half maximum (FWHM). The inferior cerebellum was used as the reference region for tau SUVr^47^ the whole cerebellum for amyloid-β^48,49^, as this have shown to obtain the best results to study AD. A quality check of the preprocessed data was be performed to check the correct preprocessing and the presence of head motion. The initial sample had *N* = 447 participants. The final sample consisted of *N* = 335 participants. We excluded 55 participants due to missing fMRI scanning parameters or belonging to an under-represented scanning facility; 39 participants had missing behavioral data, and 1 participant did not meet image quality after quality inspection).

#### Magnetic resonance imaging structural and functional preprocessing

The preprocessing procedures were adapted from Diez et al. ^50^. FMRIB Software Library v6.0.4 (FSL) and MATLAB 2021b were used for these analyses. The anatomical T1-weighted MRI preprocessing pipeline included: re-orientation to right-posterior-inferior (RPI); alignment to anterior and posterior commissures; skull stripping; gray matter, white matter and cerebrospinal fluid segmentation; and computation of non-linear transformation between individual skull-stripped T1 and 3 mm resolution MNI152 template images. The functional MRI preprocessing pipeline included: slice time correction; re-orientation to RPI; re-aligning functional volumes with a rigid body transformations (6 parameters linear transformation); computation of the transformation between individual skull-stripped T1 and mean functional images; intensity normalization; removal of confounding factors from the data using linear regression - including 12 motion-related covariates (rigid motion parameters and its derivatives), linear and quadratic terms, and five components each from the lateral ventricles and white matter. Functional images were normalized with an isotropic Gaussian kernel of 8-mm FWHM. Band-pass filtering (0.01–0.08 Hz) to reduce low-frequency drift and high-frequency noise was applied. Head motion was quantified using realignment parameters obtained during image preprocessing, which included 3 translation and 3 rotation estimates. Scrubbing of time points with excess head motion interpolated all time points with a frame displacement > 0.5 mm^51^. We used 170 time points (8 minutes and 30 seconds) not exceeding the displacement threshold in all the individuals to generate the connectivity matrices. The distributions of the correlations across time series were inspected for possible noise contamination. The initial sample had *N* = 1278 participants. The final sample consisted of *n* = 713 participants. We excluded 480 participants due to missing scanning parameters, 11 participants who did not meet T1 image quality after visual inspection, and 74 participants who did not meet functional image quality after inspection). We used the Combat harmonization algorithm to reduce the inter-scanner variability (i.e., changes in field strength, gradient nonlinearity, subject positioning, and longitudinal drift, etc.) in fMRI analyses^52,53^. We applied harmonization to fMRI-seed and degree centrality maps before statistical analyses.

### Characterizing brain imaging phenotype of unawareness

#### Distinct pattern of AD pathology in unawareness

We conducted an analysis of variance (ANCOVA) for each imaging modality. All analyses were done at the whole-brain voxel-wise level (for an overview of the imaging analysis, see Fig. 1b). We compared each subgroup against the rest, making the following comparisons: (i) aware group > control group; (ii) unaware group > control group; iii) complainer group > control group; (iv) unaware group > aware group; (v) aware group > complainer group, and (vi) unaware group > complainer group). The methods used also allowed to investigate the possible results of the reverse contrasts. Analysis included age, sex, years of education and MMSE as control covariates. MMSE was included as a covariate to control for cognition and isolate the awareness effect.

#### Possible tau spreading, amyloid progression, and functional network vulnerability

Subsequent analyses consisted in studying tau and amyloid connectivity-based spreading from the vulnerable regions found in the omnibus analysis and in finding a characterizing pattern of network organization linked to unawareness (tau spreading: Fig. 1c and d). PET-connectivity is based on the estimation of covariation in measures (amyloid or tau) across subjects^54,55^; in this sense, connectivity is measuring the relationship of increased presence of amyloid or tau between different brain regions. We used Matlab to delimit five regions of interest (ROIs) for tau-PET and two for amyloid-PET. The ROIs corresponded to peak maxima deposition in the clusters resulting from the unaware>aware comparison for each modality (we used the results that were corrected for multiple comparisons using a cluster-wise Monte Carlo simulation method; please see below in *Multiple comparisons corrections in imaging analysis*). The ROIs size was 1mm^3^, using the automatic anatomical atlas (https://www.gin.cnrs.fr/en/tools/aal/). Thus, for tau-PET connectivity, we defined the following ROIs: *i*) precuneus, *ii*) posterior cingulate cortex (PCC), *iii*) fusiform gyrus, *iv*) lateral occipital cortex, *v*) lingual gyrus (Supplementary Table 1a). For amyloid-PET connectivity, we delimited two ROIs: *i*) medial prefrontal cortex (mPFC), *ii*) medial orbitofrontal cortex (mOFC) (Supplementary Table 1b). For investigating possible routes of tau spreading or the amyloid progression, we conducted ROI to whole-brain regression analysis to find the propagation pathways from the vulnerable brain regions. Second, we performed whole-brain voxel-wise FC MRI analysis from the previous-step resulting maps, which were corrected for multiple comparisons. In both analyses, we used the control group data to describe the most probable routes of propagation and connectivity projections. The rationale for using the control group data is that this group represents an unimpaired population, and that this data was independent - from the contrast image *unaware>aware* - from where the ROIs were derived. Additionally, this approach inspects the similarity between imaging modalities and the correspondence between metabolic amyloid- or tau-propagation and functional network vulnerability. Finally, for the ROIs producing statistically significant results, we did a region-wise analysis with the aware and unaware group data in the fMRI sample. Then, we performed planned comparisons in FC differences linked to unawareness: (i) unaware group > aware group; (ii) unaware > control group. These analyses aimed to detect altered functional connectivity (FC) networks in the unaware group compared to control and aware participants.

#### Degree centrality analysis

Weighted degree (WD) analysis on rs-fcMRI data was done to identify hyper-connected patterns and between-groups spatiotemporal differences in the awareness network organization. We calculated a voxel-wise FC adjacency matrix for each participant. Only voxels corresponding to gray matter tissue were used. We obtained connectivity matrices by calculating the Pearson product-moment correlation coefficients between the time course of each pair voxels. Only the positive correlations were retained to eliminate deleterious associations between voxels due to the ambiguity of negative correlations^56,57^. The WD of each voxel was computed by summing the weights of all its connections. Individual WD maps were used for between-group comparisons. Using a General Linear Model analysis, we obtained the brain map of the unaware > aware group. Subsequently, to investigate the patterns of co-location between elevated tau or amyloid and WD, we calculated the z-scores of the unaware vs. aware comparison of each map (tau, amyloid, or WD). Finally, z-score maps were projected jointly to create an overlap of two maps: i) tau and WD; ii) amyloid and WD.

### Statistics and reproducibility

For behavioral analysis, we used Cramer-von Mises normality tests to assess sample distributions. We employed Kruskal-Wallis’ tests with post-hoc Dunn’s test for non-normally distributed variables and ANOVA with post-hoc Tukey’s and Dunnett’s tests for normally distributed variables to evaluate group differences. To estimate effect sizes, we used epsilon-squared and eta-squared. Sample sizes can be found in text, figures, and table captions. The group differences analyses used the Benjamini-Hochberg and Bonferroni methods for multiple comparisons correction. All imaging analysis results (from amyloid-PET, tau-PET, rs-fcMRI, and weighted degree rs-fcMRI images) were also corrected for multiple comparisons using a cluster-wise Monte Carlo simulation method, with 10,000 iterations to estimate the probability of false-positive clusters with a two-tailed *p*-value < 0.05 (3dClustSim; AFNI: https://afni.nimh.nih.gov/).

## Results

### Unawareness neurocognitive profile

In omnibus tests, we found between-group differences in age, years of education and MMSE (a trend in the PET sample in MMSE). Also, the overall statistical models show group differences in objective and subjective memory measurements in both samples (see Table 1 for the PET sample and Table 2 for the fMRI sample). Concerning the memory scores, in both samples, we found that the aware and unaware groups perform worse than the control group in neuropsychological memory tests. These results confirm the presence of objective memory decline in the aware and unaware groups, while the complainer group did not differ from the control group, indicating unimpaired memory performance. In relation to subjective memory complaints, we found, in both samples, that the unaware group did not differ from the control group but was significantly different compared to the aware group, which confirms the lack of awareness of memory decline in the unaware group. Note that differences in MACQ in the aware group, compared to the control group, mean that the former participants recognize their memory decline. In contrast, the controls did not have any memory impairment to admit. Even though it is not the object of this study, we found that the complainer group had similar results in objective memory tests when compared to controls (i.e., memory is not impaired) and equivalent results in MACQ when compared to the aware group (i.e., the complainer group believe they have objective memory loss despite performing within normal range on these tests). All post-hoc between-group comparisons analyses results have been reported in Table 3 for the PET sample and Table 4 for the fMRI sample. Additional Dunnett’s test analysis results confirm the post-hoc test for FCSRT score are reported in Supplementary Table 2.

**Table 3:** Post-hoc analyses results for the PET sample (*N* = 335 individuals) after omnibus between-group comparisons, investigating the differences in objective or subjective memory scores

	LM		Free FCSRT		Cued FCSRT		MACQ	
	Difference	Adjusted *p*-value [95%CI]	Difference	Adjusted *p*-value [95%CI]	Difference	Adjusted *p*-value [95%CI]	Difference	Adjusted *p*-value [95%CI]
Aware vs. complainer	−71.52	1.40e−06*** [−107.98, −35.06]	−8.57	<2.00e−16*** [−10, −7.15]	8.02	<2.00e−16*** [6.64, 9.41]	4.2	0.856 [−32.18, 40.59]
Aware vs. control	−69.21	2.00e−05*** [−109.77, −28.65]	−8.99	<2.00e−16*** [−10.56, −7.42]	8.42	<2.00e−16*** [6.89, 9.95]	171.23	<2.00e−16*** [130.76, 211.7]
Aware vs. unaware	−23.57	0.35 [−82.67, 35.53]	1.26	0.34 [−0.93, 3.45]	−1.23	−3.36 [0.9, 0.33]	167.27	1.10e−13*** [108.3, 226.24]
Complainer vs. control	2.31	0.86 [−31.96, 36.57]	−0.42	0.81 [−1.74, 0.91]	0.4	0.82 [−0.89, 1.69]	167.03	<2.00e−16*** [132.84, 201.22]
Complainer vs. unaware	47.95	0.04* [−7.02, 102.92]	9.83	<2.00e−16*** [7.8, 11.87]	−9.26	<2.00e−16*** [−11.24, −7.27]	163.06	<8.80e−15*** [108.21, 217.91]
Unaware vs. Control	−45.64	0.06 [103.41, −12.13]	−10.25	<2.00e−16*** [−12.48, −8.01]	9.66	<2.00e−16*** [7.48, 11.84]	3.97	0.856 [53.68, 61.61]

Legend: *p < 0.05, **p < 0.005, ***p < 0.001.

**Table 4:** Post-hoc analyses results for the fMRI sample (*N* = 713 individuals) after omnibus between-group comparisons, investigating the differences in objective or subjective memory scores

	LM		Free FCSRT		Cued FCSRT		MACQ	
	Difference	Adjusted *p*-value [95%CI]	Difference	Adjusted *p*-value [95%CI]	Difference	Adjusted *p*-value [95%CI]	Difference	Adjusted *p*-value [95%CI]
Aware vs. complainer	165.54	5.90e−14*** [−222.62, −108.47]	−7.98	<2.00e−16*** [−9.05, −6.92]	7.51	<2.00e−16*** [6.47, 8.54]	25.09	0.295 [−31.95, 82.13]
Aware vs. control	91.58	3.80e−16*** [−252.05, −131.11]	−7.88	<2.00e−16*** [−9.01, −6.76]	7.39	<2.00e−16*** [6.3, 8.48]	375.33	<2.00e−16*** [314.9, 435.77]
Aware vs. unaware	24.79	0.4075 [−103.76, 54.18]	0.06	1 [−1.36, 1.48]	−0.12	−1.5 [1.25, 0.99]	370.23	<2.00e−16*** [301.61, 398.87]
Complainer vs. control	26.04	0.1896 [−74.69, 22.62]	0.1	0.99 [−0.81, 1.01]	−0.12	0.98 [−1, 0.76]	350.24	<2.00e−16*** [291.3, 449.15]
Complainer vs. unaware	140.75	1.90e−07*** [70.41, 211.08]	8.05	<2.00e−16*** [6.73, 9.36]	−7.63	<2.00e−16*** [8.9, 6.36]	345.14	<2.00e−16*** [274.84, 415.43]
Unaware vs. Control	166.79	3.50e−09*** [93.67, 239.9]	−7.95	<2.00e−16*** [−9.31, −6.58]	−7.51	<2.00e−16*** [8.83, 6.19]	5.1	0.854 [−78.18, 67.97]

Legend: *p < 0.05, **p < 0.005, ***p < 0.001.

### Distinct pattern of AD pathology in unawareness

**Fig. 2:**
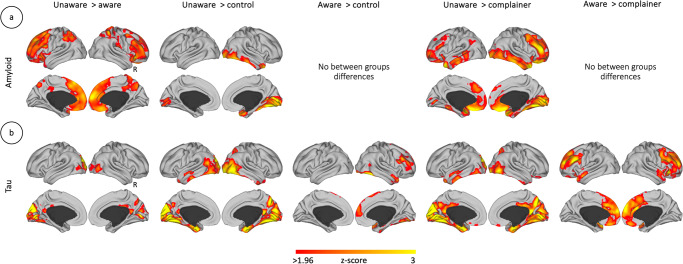
Molecular brain phenotype of unawareness in the context of AD. Between groups comparison of the (**a**) amyloid and (**b**) tau deposition (aware group *n* = 72 individuals, unaware group *n* = 25 individuals, complainer group *n* = 141 individuals and control group *n* = 87 individuals). Brain maps show results corrected for multiple comparisons. Color bars represent z-scores, with a minimum critical value or z-score of 1.96 (two-tailed 95% confidence interval). Maps are projected onto lateral and medial sections. R: Right hemisphere.

The analyses aimed to find distinctive patterns of amyloid and tau deposition linked to unawareness, compared to being aware of one’s memory decline, subjective memory complaints, or to controls (Fig. 2). We found that the unaware group had increased amyloid deposition when compared to the aware, complainer and the control groups (*p*-value < 0.05 corrected for multiple comparisons; Fig. 2a). The reverse contrasts did not produce significant results. That is, we found statistically significant differences between the participants with unawareness and aware participants which were located in areas that resemble the DMN. Specifically, the unaware participants had increased amyloid burden in the medial anterior prefrontal cortex and medial orbitofrontal regions, including inferior lateral and medial parietal areas, with visible differences in the posterior cingulate cortex and the precuneus. The orbitofrontal and lateral prefrontal cortex were also prominent when comparing the unaware group to the complainer group, as well as the lateral and medial inferior temporal gyrus towards the medial occipital cortex, including the fusiform and the lingual gyri and the calcarine sulcus. The lateral and medial occipito-temporal brain regions showed the most prominent differences when comparing the unaware group to the control group. In relation to tau, the unaware group also had increased tau deposition when compared to the rest of the groups separately (*p*-value < 0.05 corrected for multiple comparisons; Fig. 2b). The reverse contrasts did not produce significant results. Compared to the aware group, participants with unawareness had significantly increased tau deposition in the posterior cingulate cortex, precuneus, fusiform gyrus, lingual gyrus, and lateral occipital gyrus. Compared to the control participants or complainer participants, this increased tau burden in posterior DMN areas and medial visual cortex was more noticeable, but anterior to posterior MTL showed higher tau deposits. Interestingly, the aware participants displayed more tau depositions than the control and the complainer groups separately but had no differences in regard to amyloid burden (*p*-value < 0.05 corrected for multiple comparisons). Moreover, the pattern of tau depositions in the aware group shows brain pathology located in anterior brain regions (e.g., lateral PFC and anterior medial PFC, including anterior cingulate gyrus), while the unaware group showed more predominance for posterior cortical tau burden.

### Possible tau spreading, amyloid progression, and functional networks vulnerability in unawareness

**Fig. 3:**
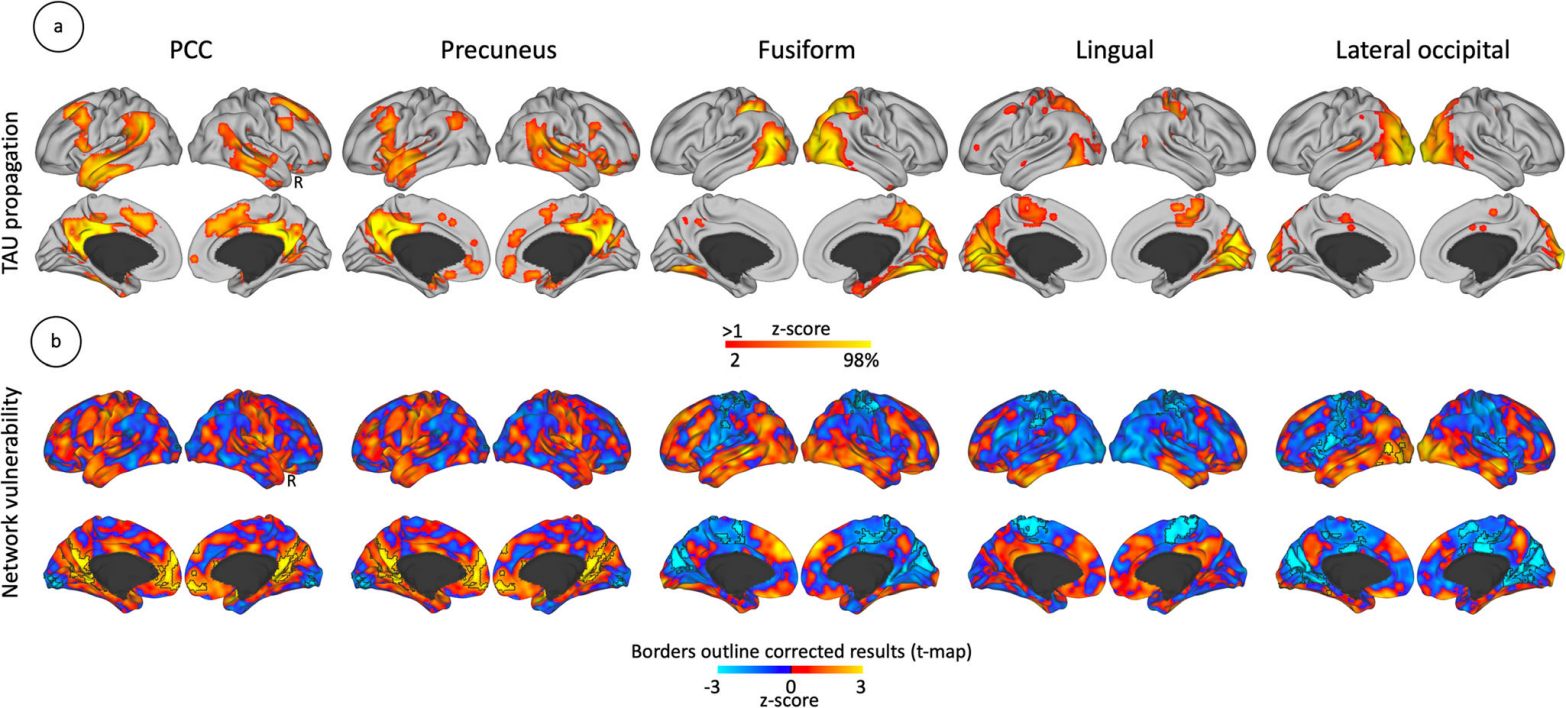
Tau spreading pathways in unawareness. **a** Probable tau spreading from brain regions that have increased tau deposition in unawareness. **b** Functional connectivity describes the brain networks vulnerable to unawareness. We used the imaging data of the control group of the PET sample (*n* = 87 individuals) for **3a** and the imaging data of the control group of the fMRI sample (*n* = 212 individuals) for **3b**. Color bars represent z-scores. The outline border shows the results corrected for multiple comparisons (two-tailed 95% confidence interval). Maps are projected onto lateral and medial sections. R: Right hemisphere.

After studying the general organization pattern of AD pathology, we centered our investigations on possible spreading trajectories of tau and amyloid deposits from vulnerable regions of the unaware phenotype (unaware > aware PET comparison) to characterize the network organization linked to unawareness. First, we used the connectivity data from the control group data to establish imaging templates of potential pathology spreading pathways in the cortical mantle using the max peaks of the unaware > aware PET changes. The controls group offers a scenario of an unbiased brain in which to make predictions of functional connectivity-based pathology progression. In the case of amyloid (see Supplementary Fig. 2), only the medial orbitofrontal cortex (mOFC) showed significant pathways comparing unaware > aware. These connections extend through the medial and lateral prefrontal cortex, including the anterior cingulate cortex, also, anterior temporal cortex, inferior parietal cortex, posterior cingulate cortex, and precuneus (Supplementary Fig. 2a). In relation to functional network pathways, we found hyperconnectivity in midline areas, locally at mOFC, and in spatially distributed areas such as inferior temporal and occipital cortices (Supplementary Fig. 2b). In relation to tau propagation pathways from key ROIs of the unaware > aware phenotype showed significant connectivity toward cortical distributed areas, heavily represented by DMN regions, as well as connectivity in the lateral and medial occipito-parietal cortex (Fig. 3a). In relation to functional network pathways (Fig. 3b), we found midline local and distributed areas at risk of connectivity disturbance. Specifically, we found hyperconnectivity in the posterior cingulate and anterior prefrontal cortex when investigating the connectivity of PCC and precuneus. As seen with probable tau spreading, this network distribution is closely related to the DMN midline structures. On the other hand, visual ROIs indicated hypoconnectivity in medial visual areas, dorsal anterior cingulate cortex and medial precentral and postcentral gyrus, and lateral precentral gyrus extending to the ventrolateral prefrontal cortex.

**Fig. 4:**
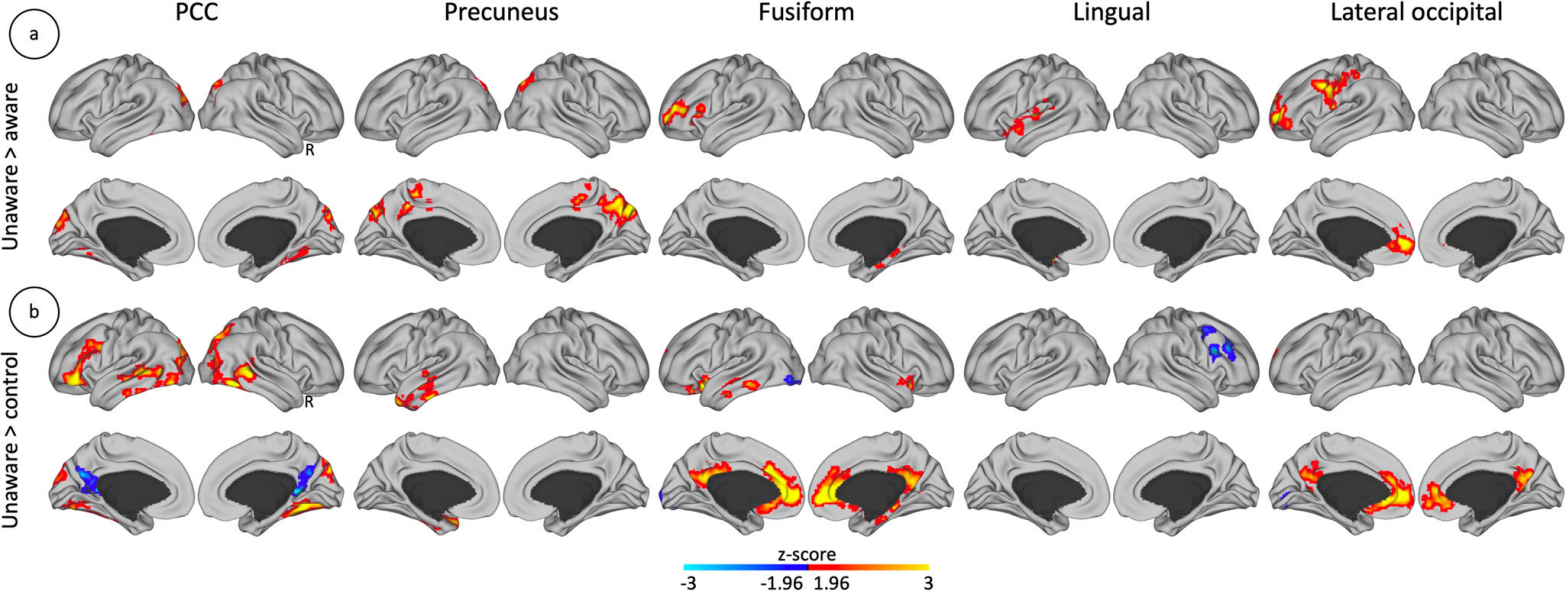
Tau-related altered FC organization in unawareness. **a** Unaware participants (*n* = 74 individuals) compared to aware participants (*n* = 129 individuals). **b** Unaware participants (*n* = 74 individuals) compared to control participants (*n* = 212 individuals). Brain maps show results corrected for multiple comparisons. Color bars represent z-scores, with a minimum critical value or z-score of 1.96 (two-tailed 95% confidence interval). Maps are projected onto lateral and medial sections. R: Right hemisphere. We used the imaging data of the aware and unaware groups of the fMRI sample.

To gain insight into the altered functional connectivity organization in unawareness, we conducted seed-based group-level comparisons using the tau-derived ROIs or the amyloid-derived ROIs of the unaware > aware phenotype. In relation to tau, compared to aware participants, unaware participants have increased local connectivity in the precuneus (i.e., increased FC spatially close to the ROI from where the analysis has been initiated). Also, the unaware participants have increased distributed connectivity in, prominently, anterior medial prefrontal cortex and ventrolateral prefrontal cortex, and in lateral occipito-temporal areas, MTL, superior and middle temporal gyrus, temporal pole, precentral gyrus, middle frontal gyrus, and frontal pole – compared to aware or to control participants (i.e., increased FC spatially far from the ROI) (Figs. 4a and b). The same analysis conducted with the amyloid-derived ROI (i.e., mOFC), these analyses yielded no significant differences between unaware and aware participants or unaware and control participants.

**Fig. 5:**
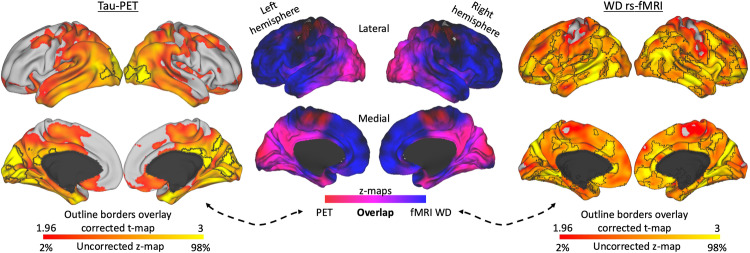
Brain systems alterations in unawareness associated with tau pathology. Unaware participants show higher tau deposition and weighted degree when compared to aware participants (*n* = 72 individuals). To associate and discriminate tau and resting-state functional connectivity effects, these results were merged in an overlap projection that revealed prominent posterior tau deposition and posterior-to-anterior increased connections visible in the brain maps. We used the PET imaging data of the aware group (*n* = 72 individuals) and unaware group (*n* = 25 individuals) for the tau deposition comparison and the fMRI data of the aware group (*n* = 129 individuals) and unaware group (*n* = 74 individuals) for the WD comparison. Brain maps show results corrected for multiple comparisons. Color bars represent z-scores; uncorrected values cover the range from 2% to 98%, while corrected values are within a minimum critical value or z-score of 1.96 (two-tailed 95% confidence interval) and are delimited by black outline borders. Maps are projected onto lateral and medial sections.

**Fig. 6:**
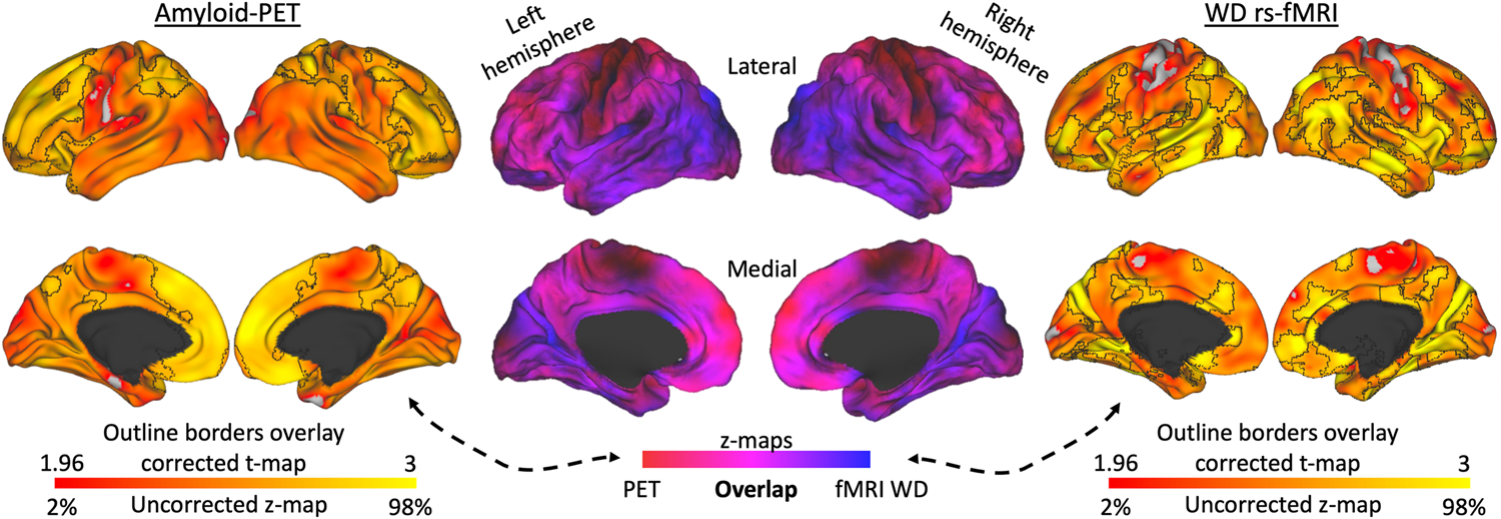
Brain systems alterations in unawareness associated with amyloid pathology. Unaware participants show higher amyloid accumulation in the anterior medial prefrontal cortex and, to a lesser extent, dorsolateral prefrontal cortex and superior parietal gyrus. To associate and discriminate amyloid and resting-state functional connectivity effects, these results were merged in an overlap projection that revealed prominent anterior amyloid deposition in coincidence with increased anterior connectivity. In contrast, posterior-lateral changes in connectivity were not associated with elevated amyloid deposition. We used the PET imaging data of the aware group (*n* = 72 individuals) and unaware group (*n* = 25 individuals) for the amyloid deposition comparison and the fMRI data of the aware group (*n* = 129 individuals) and unaware group (*n* = 74 individuals) for the WD comparison. Brain maps show results corrected for multiple comparisons. Color bars represent z-scores; uncorrected values cover the range from 2% to 98%, while corrected values are within a minimum critical value or z-score of 1.96 (two-tailed 95% confidence interval) and are delimited by black outline borders. Maps are projected onto lateral and medial sections.

Lastly, between-groups comparisons using WD maps shed some light on the connectivity hubs altered by tau or amyloid deposits of the unawareness group. Specifically, comparing the unaware to the aware participants revealed that unawareness leads to an increased number of connections; an hyperconnectivity state in temporal, parietal and occipital cortical regions (Figs. 5 and 6). Brain areas with high tau deposits show increased functional connectivity (Fig. 5), particularly in posterior cerebral areas such as the inferior medial occipital cortex (lingual gyrus), inferior and superior lateral occipital cortex, inferior temporal gyrus including the fusiform gyrus, temporooccipital region, left supramarginal gyrus and parietal operculum, right angular gyrus, posterior cingulate cortex, lateral superior temporal gyrus, left parahippocampal gyrus. Medial anterior prefrontal, lateral prefrontal, and medial parietal regions, with high amyloid deposits also co-located with brain areas showing hyperconnectivity (Fig. 6).

## Discussion

In the present study, evidence supports that AD pathophysiology and altered connectivity disruptions are present in adults with unawareness. We observed that unawareness states of memory decline associates to specific patterns of brain pathology that are unique compared to awareness and subjective memory complain individuals. We found that unawareness is characterized by increased tau deposits in midline posterior brain regions, visual medial and lateral regions, and increased amyloid deposition in midline anterior DMN. Additionally, we discovered altered network connectivity in local and distributed midline anterior and posterior brain regions, as well as lateral prefrontal and temporo-occipital regions, indicating a multi-systemic yet precise functional decline in these subjects, with a robust predominance for the cortical posterior areas of the human brain. Overall, these results extend previous research^18,23–28,33,36,37^ and support that in unawareness the spatial intersection of tau spreading and functional connectivity changes is critical to understand this phenomenon in early preclinical stages, likely preluding AD progression.

AD is a neurodegenerative disease characterized by extracellular amyloid plaques, intracellular neurofibrillary tangles, and hyperphosphorylated tau^58,59^. Years before an individual is clinically diagnosed with AD; unawareness has been shown to develop in some individuals and become worse throughout the course of AD^20,60,61^. Thus, it may be considered a unique primary symptom^6,11^. Combining subjective and objective memory assessments, we detected a group of individuals with unawareness of memory decline that, at the brain level, have typical AD pathology, reinforcing that unawareness is an early behavioral indicator of brain pathology. Previous research supports that cortical hyperphosphorylated tau presence usually is co-located in areas that show atrophy and predicts system degeneration; moreover, when tau affects specific cognitive networks, domain-specific cognitive impairments follow after tau appearance^62^. Our results indicate that unaware individuals have more tau burden in posterior DMN, a cognitive system supporting the self-referential system. Therefore, brain pathology is aligned with behavioral failure of updating self-perception^1,3,5,61^.

Theoretical models of awareness and clinical observations of individuals with AD suggest how AD pathology is detrimental to different components of awareness, including anosognosia in AD (i.e., lack of insight of deficit)^63–66^. In most clinical cases, amyloid and tau are initially found in different brain areas, but both progress through the brain systems following a hierarchal pathway. In our work, unaware participants have elevated amyloid deposits in neocortical regions extending through the medial and lateral prefrontal cortex, superior parietal cortex, medial temporal cortex, inferior occipitotemporal areas, and medial occipital areas. Conversely, tau burden was primarily located in the medial and lateral occipital cortex, medial temporal lobe and inferior temporal cortex, and medial parietal regions (precuneus and posterior cingulate cortex). The distribution of amyloid and tau deposits in unaware individuals reminds us of the early stages of sporadic late-onset AD. Amyloid deposits are first observed in the neocortex, progressing to the allocortex, basal ganglia, and thalamus, and finally extending to the pons and cerebellum^67^. After tau has damaged nuclei at the brainstem, it hierarchically progresses through the medial temporal lobe, neocortical regions such as the inferior temporal cortex and medial prefrontal cortex, medial posterior brain regions (i.e., cingulate cortex and precuneus), to finally disturb primary and secondary areas^68^. A recent review indicates that brain regions usually associated with anosognosia, awareness related to episodic memory (i.e., autonoetic consciousness) and metacognition, such as the prefrontal cortex (extending from the medial frontal cortex to superior and inferior frontal gyri), medial temporal lobe, anterior and posterior cingulate cortex and, insula, are co-localized with regions representing the DMN^63^. The DMN is composed of a core system and two distinct subsystems^69^. The core system, which implicates the posterior medial parietal and anterior medial prefrontal cortex, is engaged when individuals make autobiographical decisions, either present or future self-referred judgments. In our research, unaware individuals had more tau burden in posteromedial and more amyloid deposits in anteromedial brain areas, which is compatible with the self-referential system starting to fail in preclinical AD. The dorsomedial prefrontal cortex subsystem tends to be linked to present self-referential judgments, while future decisions engage the MTL subsystem^69^. Subthreshold elevated tau and amyloid deposition in the dorsolateral prefrontal cortex, and MTL structures was also visible, implicating other DMN structures. Altogether, this pathology distribution affecting DMN core regions early in the disease supports predictions of disease progression^24^ and is aligned with previous research on the trajectory of unawareness in AD^20,24,60^, functional studies^38^ and FDG-PET studies^25,27,28^, highlighting its clinical value. Importantly, these findings may have clinical relevance because they are directly associated with the reliability of the patient’s complaints of dysfunction. Specifically, our results question the clinical use of an individual’s reports of subjective cognitive decline, as some high-risk individuals may be missed using this approach. Importantly, unawareness seems to be a direct result of pathological changes affecting the self-referential network system, or as authors have indicated, dysfunction of some aspects of consciousness might be considered a central phenomenological characteristic of AD^64^. Literature review reveals that the specific patterns of deficits related to unawareness and its correspondence to the presence of pathology in cortical brain regions can be heterogeneous across patients^64^. As such, the current findings provide further evidence that individuals unaware of subtle cognitive changes may represent a specific risk group for AD, and unawareness as a clinical trait deserves more neuroimaging research investigating the co-localization patterns between the brain correlates of behavioral changes and pathology distribution.

Theoretical accounts of anosognosia can be linked to some neuropathological changes associated with the brain phenotype of anosognosia in AD. According to the Cognitive Awareness Model (CAM)^3,5,70,71^, individuals with diminished self-awareness experience a failure in the updating processes of the personal database. This failure occurs during the monitorization of the self: the performance inputs need to be compared to the stored information, in the personal database. When the system works properly, the output of this comparison allows for an update of the personal database. If a mismatch between the new and the stored information is detected, the information is released to the metacognitive awareness system to provide consciousness of decision-making. However, loss of self-awareness impacts the proper function of the comparing mechanisms and updating the self. In this account, multiple neurocognitive factors can lead to anosognosia in AD, being mnemonic anosognosia the one related to memory or consolidation of information therefore, impacting the updating processes^72^. In CAM, anosognosia is partly explained by a loss of mnemonic ability in which knowledge about self-ability is limited to outmoded semantic understanding. Individuals with unawareness suffer a loss of information on performance judgment, a loss of recollection of personally experienced events and a trend to rely on remote and abstracted knowledge when judging self-actions. The most critical aspect linking unawareness and AD is precisely the loss of autobiographical memory and semanticization, along with the failure to update self-knowledge. This leads to a non-updated personal database. Thus, the stable representations of personal ability and the individual’s self-concept stay anchored in past experiences.

Unawareness individuals had a pattern of increased network synchronicity co-localized with increased tau deposits in posterior brain regions and increased amyloid deposits in anterior medial cortical areas. Different interpretations have been linked to hyper-connectivity in the aging brain. In the AD spectrum, when individuals show subtle cognitive decline but still do not meet the criteria for dementia, compensatory mechanisms seem to reflect a coping mechanism as pathology starts to accumulate; thus, hyper-connectivity could be reflecting unaffected neurons overexertion to perform at a similar level. Another plausible explanation is network destabilization preventing proper functioning. Either one or the other, changes in brain regions’ synchronicity appear early in AD, along with other pathophysiological hallmarks. Previous research has found that amyloid deposits are usually located within the DMN areas, such as the medial posterior parietal, medial prefrontal, lateral inferior parietal cortices, and the retrosplenial and medial temporal cortices^73^. Mixed patterns of altered FC (hyper- and hypo-connectivity) have been found in individuals with elevated amyloid burden^74^. In some experiments, there is a decreased connectivity in posterior medial areas (i.e., precuneus, PCC), ventral medial prefrontal cortex, and angular gyrus^73,75^ or medial temporal areas^76^. At the same time, other reports show an increased connectivity between dorsal and anterior medial prefrontal cortex and lateral temporal cortices in the context of preclinical AD, which might be driven by the different experimental questions (i.e., comparing amyloid positive or negative cognitively unimpaired individuals). Our work found a co-location pattern between hyper-connectivity in the medial orbitofrontal and frontal cortex and elevated amyloid deposition in unaware participants compared to aware. The relationship between cortical hyperphosphorylated tau burden and abnormal patterns of network connectivity has been less explored. Some authors point to an inverse association between tau burden and connectivity in preclinical stages^76,77^, while others find positive relations^78^. For instance, DMN and salience network hyper-connectivity in amyloid-positive individuals in the early stages of preclinical AD was related to less tau burden in the inferior temporal cortex^77^. Cerebrospinal fluid (CSF) amyloid positivity was characterized by hypo-connectivity between medial and anterior MTL regions, and that increased CSF tau level was associated with hypo-connectivity between the entorhinal, hippocampus, and posterior-medial brain regions^76^. Contrarily, positive associations have been found between the hippocampus and retrosplenial cortex pair connectivity and tau burden in the medial parietal cortex^78^. In other research^79^, when considering the relationship between amyloid, tau, and FC, two patterns were distinguished: (i) areas displaying high tau deposits were related to more hypo-connectivity in the elder compared to younger adults; (ii) areas displaying high amyloid cortical deposition were associated with hyper-connectivity in elder compared to younger adults; however, local connectivity was more sensible to these two patterns than distributed connectivity. None of these investigations took into consideration awareness of memory decline. Also, different brain analysis outcomes might be due to differences in estimating amyloid and tau, differences in methodologic approaches modeling functional networks, and differences in demographic characteristics and other risk factors (i.e., specific genes, apolipoprotein e4, family history), contributing all of them differently to developing AD^80,81^. One limitation of assessing unawareness is the variety of instruments available and the score-variability between them. We tried to minimize this effect by integrating three objective memory scores into a self-report questionnaire; however, we acknowledge our method is one of the many possibilities, and we did not include an informant report. In addition, the MACQ questionnaire investigates individuals’ self-perception of cognitive decline by using questions that target early lifetime periods (childhood). This approach could bias individuals’ responses by drawing attention to all age-related complaints. In this regard, complainers could have been classified as control individuals. Another limitation is the lack of longitudinal data to investigate changes over time. Furthermore, our cohort includes only clinically normal participants (defined as CDR = 0 and MMSE > 25, see SI), thus, we could not compare the current participants to MCI or AD participants.

The present research investigated the brain phenotype of adults at risk for developing AD presenting subtle memory decline and unawareness. These individuals show increased amyloid deposition in anterior medial and lateral prefrontal cortex, as well as increased tau deposits in posterior cortical regions. Additionally, unawareness is characterized by a hyperconnectivity pattern that highly intersect in anterior to posterior regions of the human brain, overlapping core regions of the DMN. Thus, it suggests that unawareness is a multi-faced manifestation mostly originated by tau pathology inducing hyperconnectivity changes in the posterior self-referential core system of the human brain.

### Supplementary information

Supplementary Information

Reporting Sumnmary

## Data Availability

The Anti-Amyloid Treatment in Asymptomatic Alzheimer’s (A4) and the Longitudinal Evaluation of Amyloid Risk and Neurodegeneration study (LEARN) studies are led by Dr. Sperling at Brigham and Women’s Hospital, Harvard Medical School and Dr. Aisen at the Alzheimer’s Therapeutic Research Institute (ATRI), University of Southern California. The A4 and LEARN Studies are coordinated by ATRI at the University of Southern California (https://a4study.org/). The data are publicly available through the Laboratory for Neuro Imaging at the University of Southern California upon completion of a data user agreement. Access can be obtained via this website: https://ida.loni.usc.edu/.

## Code availability

Standard behavioral and neuroimaging analysis were used to derive the results of this study. The following software was used for data analysis: R project (https://www.r-project.org/), FMRIB Software Library v6.0.4 (FSL; https://fsl.fmrib.ox.ac.uk/fsl/fslwiki/), FreeSurfer v6 (https://surfer.nmr.mgh.harvard.edu/), MATLAB 2021b, Automatic anatomical atlas (https://www.gin.cnrs.fr/en/tools/aal/), and 3dClustSim; AFNI.
