# Supplementary material for: Multi-modal Neuroimaging Phenotyping of Mnemonic Anosognosia in the Aging Brain
Source: Commun Med (Lond). 2024 Apr 5;4:65. doi: 10.1038/s43856-024-00497-9 (PMC10997795; doi:10.1038/s43856-024-00497-9)

SUPPLEMENTARY INFORMATION

Supplementary table 1. MNI coordinates of the region of interest used in PET-connectivity analysis. A) tau spreading and B) amyloid progression analyses.

	**MNI coordinates**		
	**X**	**Y**	**Z**
**A) Tau spreading**			
Posterior cingulate cortex	12	48	17
Precuneus	18	-60	30
Lingual gyrus	12	-79	0
Fusiform gyrus	30	-72	-8
Lateral occipital gyrus	-36	-96	0
**B) Amyloid progression**			
Medial frontal cortex	4	56	12
Medial orbitofrontal cortex	44	77	27

Supplementary table 2. Planned between-group comparisons for investigating objective memory differences. The post-hoc Dunnett's test was used to compute differences in free and cued FCSRT scores. Confidence interval = 95%. All three groups were compared to the control group. Legend: * p<0.05, **p<0.005, ***p<0.001.

	**Difference**	**Adjusted p-value**
PET sample - Free FCSRT		
Aware - control	-8.990	<2e-16 ***
Complainer - control	-0.415	0.811
Unaware - control	-10.248	<2e-16 ***
PET sample - Cued FCSRT		
Aware - control	8.424	<2e-16 ***
Complainer - control	0.400	0.816
Unaware - control	9.657	<2e-16 ***
fMRI sample - Free FCSRT		
Aware - control	-7.88	<2e-16 ***
Complainer - control	0.10	0.99
Unaware - control	-7.95	<2e-16 ***
fMRI sample - Cued FCSRT		
Aware - control	7.388	<2e-16 ***
Complainer - control	-0.119	0.981
Unaware - control	7.512	<2e-16 ***

Supplementary Figure 1. Neuroimaging data inclusion flow diagram. We used neuroimaging data from the Anti-Amyloid Treatment in Asymptomatic Alzheimer's Disease (A4) / Longitudinal Evaluation of Amyloid Risk and Neurodegeneration (LEARN) Study were used in this research. Our initial sample consisted of 1725 individuals. Individuals with only functional MRI data were assigned to the fMRI sample. Initially, 1278 individuals were included. After preprocessing, the final fMRI sample consisted of 713 individuals (left-hand side of the figure / blue boxes). Individuals with amyloid- and tau-PET imaging data were assigned to the PET sample. Initially, 447 individuals were included. After preprocessing, the final fMRI sample consisted of 335 individuals (right-hand side of the figure / green boxes).

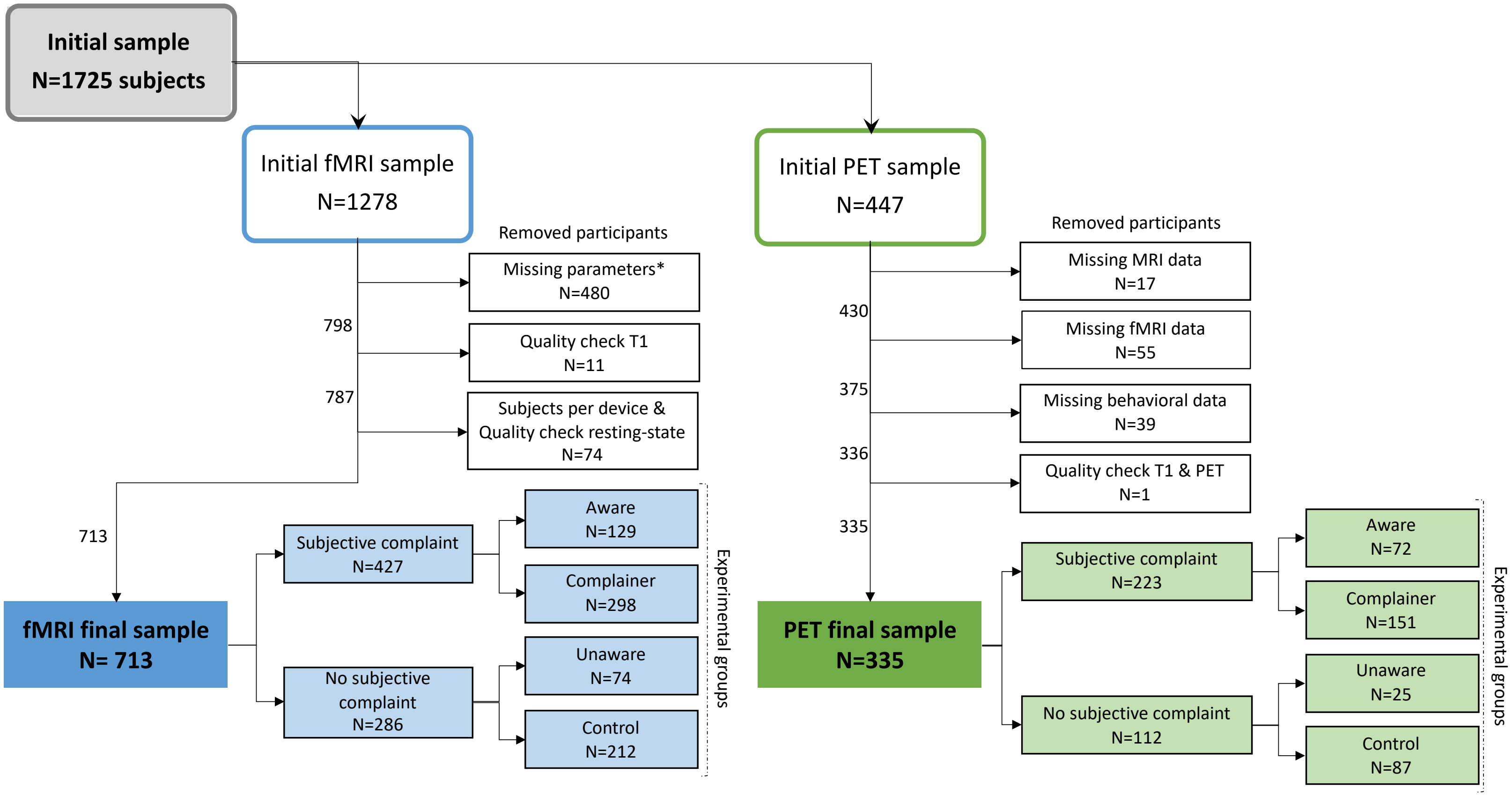

Supplementary Figure 2. Amyloid spreading pathways in unawareness and related altered FC. A) Results for PIB-connectivity analysis from the brain regions –mOFC – that have increased amyloid deposition in unaware participants compared to aware participants. B) Functional connectivity analysis results from the mOFC ROI, describing the brain networks vulnerable to unawareness. Color bars represent uncorrected results (z-scores), borders outline corrected results for multiple comparisons (two-tailed 95% confidence interval). Maps are projected onto lateral and medial sections. R: Right hemisphere. Abbreviations: mOFC: medial orbitofrontal cortex; R: Right hemisphere. We used the imaging data of the control group of the PET sample for (A) and the imaging data of the control group of the fMRI sample for (B).

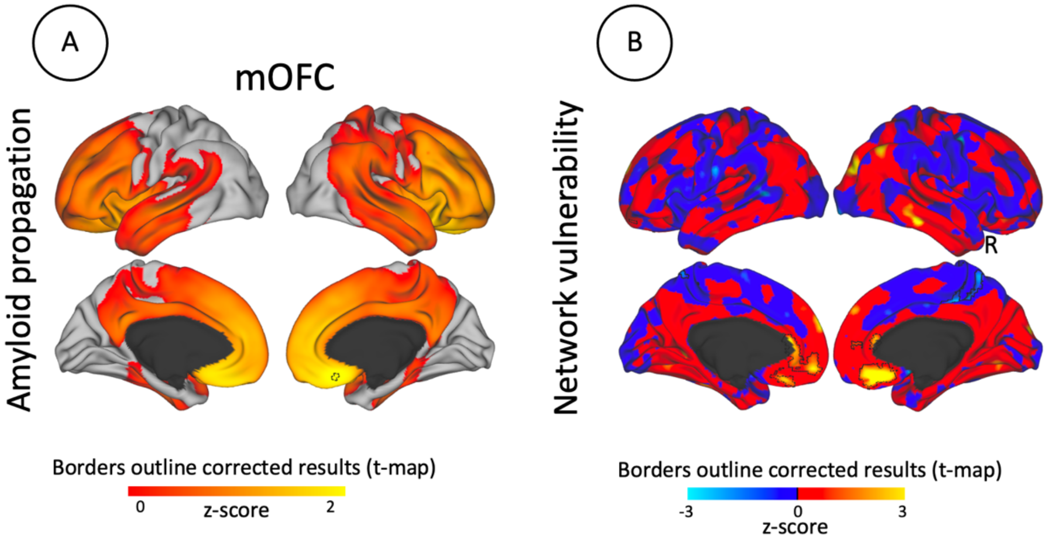

Supplement: Supplementary file 1 — Supplementary Information [file 43856_2024_497_MOESM1_ESM.pdf]
