# Supplementary material for: Multi-modal Neuroimaging Phenotyping of Mnemonic Anosognosia in the Aging Brain
Source: Commun Med (Lond). 2024 Apr 5;4:65. doi: 10.1038/s43856-024-00497-9 (PMC10997795; doi:10.1038/s43856-024-00497-9)
Supplement: Supplementary file 2 — Reporting Sumnmary [file 43856_2024_497_MOESM2_ESM.pdf]

Corresponding author(s): Jorge Sepulcre
Patrizia Vannini

Last updated by author(s): Jan 8, 2024

Reporting Summary

Nature Portfolio wishes to improve the reproducibility of the work that we publish. This form provides structure for consistency and transparency in reporting. For further information on Nature Portfolio policies, see our Editorial Policies and the Editorial Policy Checklist.

Statistics

For all statistical analyses, confirm that the following items are present in the figure legend, table legend, main text, or Methods section.

n/a	Confirmed	
☐	☒	The exact sample size (*n*) for each experimental group/condition, given as a discrete number and unit of measurement
☒	☐	A statement on whether measurements were taken from distinct samples or whether the same sample was measured repeatedly
☐	☒	The statistical test(s) used AND whether they are one- or two-sided<br>*Only common tests should be described solely by name; describe more complex techniques in the Methods section.*
☐	☒	A description of all covariates tested
☐	☒	A description of any assumptions or corrections, such as tests of normality and adjustment for multiple comparisons
☐	☒	A full description of the statistical parameters including central tendency (e.g. means) or other basic estimates (e.g. regression coefficient) AND variation (e.g. standard deviation) or associated estimates of uncertainty (e.g. confidence intervals)
☐	☒	For null hypothesis testing, the test statistic (e.g. *F*, *t*, *r*) with confidence intervals, effect sizes, degrees of freedom and *P* value noted<br>*Give P values as exact values whenever suitable.*
☒	☐	For Bayesian analysis, information on the choice of priors and Markov chain Monte Carlo settings
☒	☐	For hierarchical and complex designs, identification of the appropriate level for tests and full reporting of outcomes
☒	☐	Estimates of effect sizes (e.g. Cohen's *d*, Pearson's *r*), indicating how they were calculated

Our web collection on statistics for biologists contains articles on many of the points above.

Software and code

Policy information about availability of computer code

Data collection	The behavioral and imaging data used in this work comes from the Anti-Amyloid Treatment in Asymptomatic Alzheimer's (A4) and Longitudinal Evaluation of Amyloid Risk and Neurodegeneration (LEARN) studies (Sperling, R. A. et al. The A4 study: stopping AD before symptoms begin? Sci. Transl. Med. 6, 228fs13 (2014)). Data was retrieved from the Imaging and Data Archive (IDA) repository (https://ida.loni.usc.edu/)).
Data analysis	FMRIB Software Library v6.0.4 (FSL; https://fsl.fmrib.ox.ac.uk/fsl/fslwiki/)<br>FreeSurfer v6 (https://surfer.nmr.mgh.harvard.edu/)<br>MATLAB 2021b<br>R package (https://www.R-project.org/)<br>Automatic anatomical atlas (https://www.gin.cnrs.fr/en/tools/aal/)<br>3dClustSim; AFNI: https://afni.nimh.nih.gov/).

For manuscripts utilizing custom algorithms or software that are central to the research but not yet described in published literature, software must be made available to editors and reviewers. We strongly encourage code deposition in a community repository (e.g. GitHub). See the Nature Portfolio guidelines for submitting code & software for further information.

Data

Policy information about availability of data

All manuscripts must include a data availability statement. This statement should provide the following information, where applicable:

- Accession codes, unique identifiers, or web links for publicly available datasets
- A description of any restrictions on data availability
- For clinical datasets or third party data, please ensure that the statement adheres to our policy

In house developed code used within this manuscript will be available from the corresponding author upon request.

The A4 Study is a secondary prevention trial in preclinical Alzheimer's disease, aiming to slow cognitive decline associated with brain amyloid accumulation in clinically normal older individuals. The A4 Study is funded by a public-private-philanthropic partnership, including funding from the National Institutes of Health-National Institute on Aging, Eli Lilly and Company, Alzheimer's Association, Accelerating Medicines Partnership, GHR Foundation, an anonymous foundation and additional private donors, with in-kind support from Avid and Cogstate. The companion observational Longitudinal Evaluation of Amyloid Risk and Neurodegeneration (LEARN) Study is funded by the Alzheimer's Association and GHR Foundation. The A4 and LEARN Studies are led by Dr. Reisa Sperling at Brigham and Women's Hospital, Harvard Medical School and Dr. Paul Aisen at the Alzheimer's Therapeutic Research Institute (ATRI), University of Southern California. The A4 and LEARN Studies are coordinated by ATRI at the University of Southern California, and the data are made available through the Laboratory for Neuro Imaging at the University of Southern California. The participants screening for the A4 Study provided permission to share their de-identified data in order to advance the quest to find a successful treatment for Alzheimer's disease. We would like to acknowledge the dedication of all the participants, the site personnel, and all of the partnership team members who continue to make the A4 and LEARN Studies possible. The complete A4 Study Team list is available on: a4study.org/a4-study-team.

Human research participants

Policy information about studies involving human research participants and Sex and Gender in Research.

Reporting on sex and gender

The covariable "sex" was used in this work, in both imaging and behavioral analysis as a control variable within statistical analysis. Individuals' sex information was used according to the information available in the database (A4/LEARN data; retrieved from the Imaging and Data Archive (IDA) repository, https://ida.loni.usc.edu/). According to imaging data availability, we used two samples with four experimental groups. Sample 1 - PET imaging (N, % females): group 1 (72 (45.8%)), group 2 (25 (40%)), group 3 (151 (64.9%)), group 4 (87 (62.1%)). Sample 2 - functional MR imaging (N, % females): group 1 (129 (50.4%)), group 2 (74 (33.8%)), group 3 (298(67.8%)), group 4 (212 (69.7%)).

Population characteristics

Data used in this work comes from A4/LEARN studies. According to imaging data availability, we used two samples with four experimental groups. Demographics have been describes within the manuscript (Table 1 and Table 2).

Sample 1 - PET imaging
N (% Females): group 1: 72 (45.8%); group 2: 25 (40 %); group 3: 151 (64.9 %); group 4: 87 (62.1 %)
Mean age (SD): group 1: 73.10 (4.85); group 2: 72.98 (3.85); group 3: 70.36 (4.43); group 4: 70.75 (4.30)
Mean education (SD): group 1: 17 (2.64); group 2: 15.68 (1.84); group 3: 16.07 (2.68); group 4: 16.18 (3.22)
Mean MMSE (SD): group 1: 28.25 (1.63); group 2: 28.4 (1.54); group 3: 28.78 (1.15); group 4: 28.85 (1.21)

Sample 2 - functional MR imaging
N (% Females): group 1: 129 (50.4%); group 2: 74 (33.8%); group 3: 298 (67.8%); group 4: 212 (69.7%)
Mean age (SD): group 1: 71.99 (4.91); group 2: 72.45 (5.06); group 3: 70.89 (4.42); group 4: 70.34 (4.31)
Mean education (SD): group 1: 17.4 (2.93); group 2: 16.95 (2.7); group 3: 16.38 (2.59); group 4: 16.98 (2.73)
Mean MMSE (SD): group 1: 28.67 (1.34); group 2: 28.18 (1.45); group 3: 28.90 (1.15); group 4: 28.92 (1.19)

Original inclusion criteria for A4/LEARN studies are described in (Sperling, R. A. et al. The A4 study: stopping AD before symptoms begin? Sci. Transl. Med. 6, 228fs13 (2014)). No additional inclusion / exclusion criteria regarding population-characteristics were applied to develop this work using the A4/LEARN data.

Recruitment

Data used in this work comes from A4/LEARN studies. Participants recruitment is described in Sperling, R. A. et al. Association of Factors With Elevated Amyloid Burden in Clinically Normal Older Individuals. JAMA Neurol. 77, 735 (2020).

Ethics oversight

Institutional review board approval was secured at the participating sites.

The A4 Study is a secondary prevention trial in preclinical Alzheimer's disease, aiming to slow cognitive decline associated with brain amyloid accumulation in clinically normal older individuals. The A4 Study is funded by a public-private-philanthropic partnership, including funding from the National Institutes of Health-National Institute on Aging, Eli Lilly and Company, Alzheimer's Association, Accelerating Medicines Partnership, GHR Foundation, an anonymous foundation and additional private donors, with in-kind support from Avid and Cogstate. The companion observational Longitudinal Evaluation of Amyloid Risk and Neurodegeneration (LEARN) Study is funded by the Alzheimer's Association and GHR Foundation. The A4 and LEARN Studies are led by Dr. Reisa Sperling at Brigham and Women's Hospital, Harvard Medical School and Dr. Paul Aisen at the Alzheimer's Therapeutic Research Institute (ATRI), University of Southern California. The A4 and LEARN Studies are coordinated by ATRI at the University of Southern California, and the data are made available through the Laboratory for Neuro Imaging at the University of Southern California.

Note that full information on the approval of the study protocol must also be provided in the manuscript.

Field-specific reporting

Please select the one below that is the best fit for your research. If you are not sure, read the appropriate sections before making your selection.

☒ Life sciences ☐ Behavioural & social sciences ☐ Ecological, evolutionary & environmental sciences

For a reference copy of the document with all sections, see nature.com/documents/nr-reporting-summary-flat.pdf

Life sciences study design

All studies must disclose on these points even when the disclosure is negative.

Sample size	Imaging data of 1,725 participants from the Anti-Amyloid Treatment in Asymptomatic Alzheimer's (A4) and Longitudinal Evaluation of Amyloid Risk and Neurodegeneration (LEARN)
Data exclusions	After imaging data preprocessing, some individuals had to be removed from each of the two samples. Please see below:<br><br>PET sample: The final sample consisted in N=335 participants (55 participants had missing MRI/fMRI data, 39 participants had missing behavioral data and 1 participant did not meet image quality after visual inspection).<br><br>fMRI sample: The final sample consisted in n=713 participants (480 participants had missing scanning parameters, 11 participants did not meet T1 image quality after visual inspection, 74 participants did not meet functional image quality after visual inspection).
Replication	The data used in this work is unique. Replication analysis within imaging modalities have not been conducted because we lack of samples with the same data availability (amyloid-PET, tau-PET and MRI with the same awareness reports).
Randomization	Four subgroups were defined to investigate the brain phenotypic characteristic of adults with mnemonic anosognosia (aware, unaware, subjective complainer, and control groups; Figure 1A). Classification of participants into groups was based on objective and subjective memory assessments. We used neuropsychological tests to assess participants' performance on memory system and to evaluate objective memory decline: the free and cued selective reminding (FCSRT) test 39 and the logical memory (LM) test. A participant was classified as having memory decline when performance in both tests was below population norms. The delayed score from the LM assesses episodic memory and is used in combination with years of education to determine memory impairments (a score equal to or less than 8 for 16 years of education, a score of 4 for 8 to 15 years of education, and a score of 2 for 0 to 7 years of education). FCSRT offers a controlled learning setting to a reliable metric that englobes episodic memory encoding, recording, and retrieving processes. A score equal to or inferior to 24 in free recall and 44 in total recall in FCSRT indicates objective memory problems. For assessing subjective memory performance, the Memory Assessment Clinic Questionnaire (MACQ) 40 was used. The MACQ is a brief self-reported questionnaire composed by 5 questions, like: "recalling where you have put objects (such as keys) in your home or office" or "remembering specific facts from a newspaper or magazine article you have just finished reading". Participants must reply to these questions in relation to when they were in high school or college ("as compared to when you were in high school or college, how would you describe your ability to perform the following tasks involving your memory"). The questionnaire includes a final and more general question: "in general, how would you describe your memory as compared to when you were in high school". For all 6 questions, it uses a likert-rating scale (i.e., "much better now", "somewhat better now", "about the same", "somewhat poorer now", "much poorer now"). The MACQ generates a score (range 7-35) that quantifies degree of memory complaint. Overall, this questionnaire targets age-related changes in that the subject is asked to rate current abilities compared to past abilities 40. A participant is considered to have subjective memory complaints when the score is equal to or superior to 25 40. Subsequently, the aware group is characterized by having both memory complaints and objective memory impairments. Mnemonic anosognosia, or unaware group, has objective memory loss, but these participants do not demonstrate subjective memory complains. Contrary to the unaware group, the complainer group shows subjective memory complaints but does not have objective memory impairments. Finally, the control group does not have subjective or objective memory problems. In this study, we focus on investigating the neurobehavioral characteristics of the unaware group, which shows mnemonic anosognosia, compared to the aware, the complainer, and the control group, which cover the spectrum of possible aging profiles.
Blinding	The authors that conducted the research did not participate in data collection. For the purpose of this work, the authors divided the participants in four groups that allowed to compare the experimental groups between them.

Reporting for specific materials, systems and methods

We require information from authors about some types of materials, experimental systems and methods used in many studies. Here, indicate whether each material, system or method listed is relevant to your study. If you are not sure if a list item applies to your research, read the appropriate section before selecting a response.

Materials & experimental systems

n/a	Involved in the study
☒	☐ Antibodies
☒	☐ Eukaryotic cell lines
☒	☐ Palaeontology and archaeology
☒	☐ Animals and other organisms
☒	☐ Clinical data
☒	☐ Dual use research of concern

Methods

n/a	Involved in the study
☒	☐ ChIP-seq
☒	☐ Flow cytometry
☐	☒ MRI-based neuroimaging

Magnetic resonance imaging

Experimental design

Design type	Resting-state
Design specifications	One structural and one functional resting-state scan per participant.
Behavioral performance measures	n/a

Acquisition

Imaging type(s)	Structural and functional
Field strength	3T
Sequence & imaging parameters	Resting state functional MRI was used to measure changes in blood oxygenation level dependent (BOLD) T2* signal while the participants remained still with their eyes open. Gradient echo or gradient echo planar imaging (EPI) sequences with the following parameters were acquired: 3000 ms TR; 30 ms TE; 80- or 90-degrees flip angle; 3 mm isotropic voxels.
Area of acquisition	Whole brain

Diffusion MRI ☐ Used ☒ Not used

Preprocessing

Preprocessing software	FMRIB Software Library v6.0.4 (FSL) and MATLAB 2021b.
Normalization	Non-linear transformation between individual skull-stripped T1 and 3 mm resolution MNI152 template images.
Normalization template	MNI space. We used the 3 mm resolution MNI152 template.
Noise and artifact removal	Removal of confounding factors from the data using linear regression - including 12 motion-related covariates (rigid motion parameters and its derivatives), linear and quadratic terms, and five components each from the lateral ventricles and white matter.<br>The distributions of the correlations across time series were inspected for possible noise contamination.
Volume censoring	Head motion was quantified using realignment parameters obtained during image preprocessing, which included 3 translation and 3 rotation estimates. Scrubbing of time points with excess head motion interpolated all time points with a frame displacement > 0.5 mm 64. We used 170 time points (8 minutes and 30 seconds) not exceeding the displacement threshold in all the individuals to generate the connectivity matrices.

Statistical modeling & inference

Model type and settings	General Linear Models
Effect(s) tested	We conducted an analysis of variance (ANCOVA) for each imaging modality. We compared each subgroup against the rest, making the following comparisons: i) aware group > control group; ii) unaware group > control group; iii) complainer group > control group; iv) unaware group > aware group; v) aware group > complainer group, and vi) unaware group > complainer group). The reverse contrasts were also tested. The methods used also allowed to investigate the possible results of the reverse contrasts. Analysis included age, sex, years of education and MMSE as adjusted covariates. MMSE was included as a covariate to control for cognition and isolate the awareness effect.

Specify type of analysis: ☐ Whole brain ☐ ROI-based ☒ Both

Anatomical location(s)	Please see below the l MNI coordinates (X,Y,Z) of the anatomical locations.<br><br>Tau spreading<br>Posterior cingulate cortex 12, 48, 17<br>Precuneus 18, -60, 30<br>Lingual gyrus 12, -79, 0<br>Fusiform gyrus 30, -72, -8<br>Lateral occipital gyrus -36, -96, 0<br><br>Amyloid progression<br>Medial frontal cortex 4, 56, 12<br>Medial orbitofrontal cortex 44, 77, 27
Statistic type for inference<br>(See Eklund et al. 2016)	Cluster-wise. Monte Carlo simulation method, with 10,000 iterations to estimate the probability of false-positive clusters with a two-tailed p-value<0.05 (3dClustSim; AFNI: https://afni.nimh.nih.gov/).
Correction	All imaging analysis results (amyloid-PET, tau-PET, rs-fcMRI, and weighted degree rs-fcMRI) were corrected for multiple comparisons using a cluster-wise Monte Carlo simulation method, with 10,000 iterations to estimate the probability of false-positive clusters with a two-tailed p-value<0.05 (3dClustSim; AFNI: https://afni.nimh.nih.gov/).

Models & analysis

n/a	Involved in the study
☐	☒ Functional and/or effective connectivity
☐	☒ Graph analysis
☒	☐ Multivariate modeling or predictive analysis

Functional and/or effective connectivity	We did region-wise functional connectivity analysis on resting state functional MRI data. The region-wise FC analysis departed from the anatomical locations (regions of interest) found in the PET analysis. Individual FC maps were used for between-group comparisons using General Lineal Model analyses.
Graph analysis	Degree centrality analysis. Weighted degree analysis on rs-fcMRI data were done. We calculated a voxel-wise FC adjacency matrix for each participant. Only voxels corresponding to gray matter tissue were used. We obtained connectivity matrices by calculating the Pearson product-moment correlation coefficients between the time course of each pair voxels (voxel-size 3 mm cubic-milimeter). Only the positive correlations were retained to eliminate deleterious associations between voxels due to the ambiguity of negative correlations. The degree centrality of each voxel was computed by summing the weights of all its connections. Individual degree centrality maps were used for between-group comparisons using General Lineal Model analyses.
